# Supplementary material for: Endogenous bioluminescent reporters reveal a sustained increase in utrophin gene expression upon EZH2 and ERK1/2 inhibition
Source: Commun Biol. 2023 Mar 25;6:318. doi: 10.1038/s42003-023-04666-9 (PMC10039851; doi:10.1038/s42003-023-04666-9)
Supplement: Supplementary file 2 — Supplementary Information [file 42003_2023_4666_MOESM2_ESM.pdf]

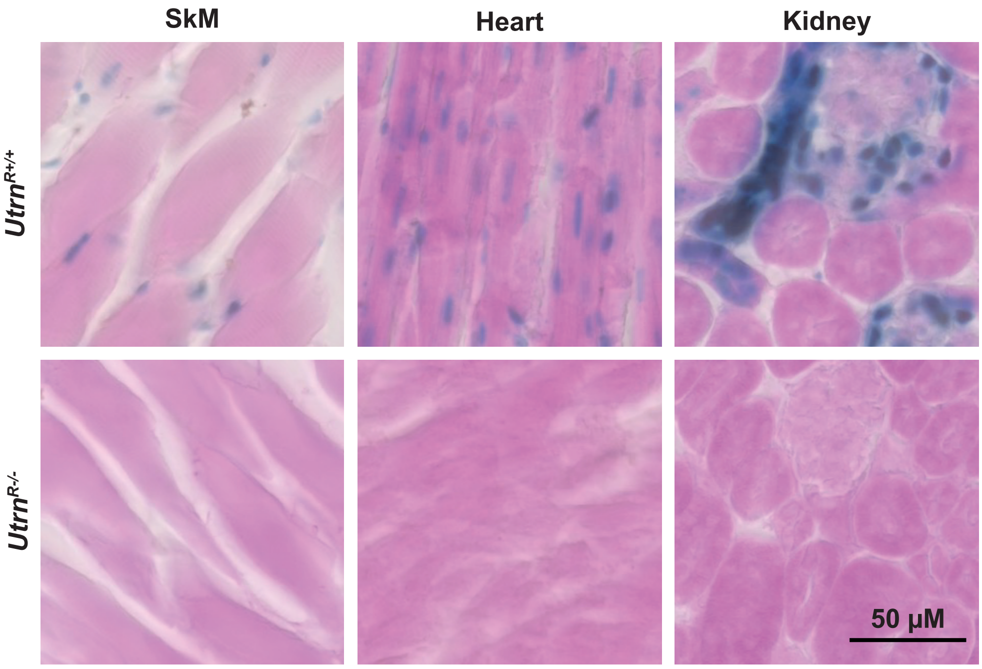
SkM
Heart
Kidney
Utrn R+/+
Utrn R-/-
50 μM

Supplementary Figure 1: Visualising expression of *Utrn* using a *lacZ* reporter

Related to Figure 1

X-Gal staining (blue) of adult tissue sections from the extensor digitorum longus (EDL) skeletal muscle (SkM), heart and kidney of reporter ($Utrn^{R+/+}$) and WT ($Utrn^{R-/-}$) mice. Sections were countered stained with Nuclear Fast Red (red/pink). In the $Utrn^{R+/+}$ samples, signal (blue) was highest in the podocytes of the glomerulus in the kidney, lower in cardiac muscle, and in skeletal muscle (SkM) signal was only evident in nuclei external to the myofibres. No *lacZ* signal was detected in the WT $Utrn^{R-/-}$ samples.

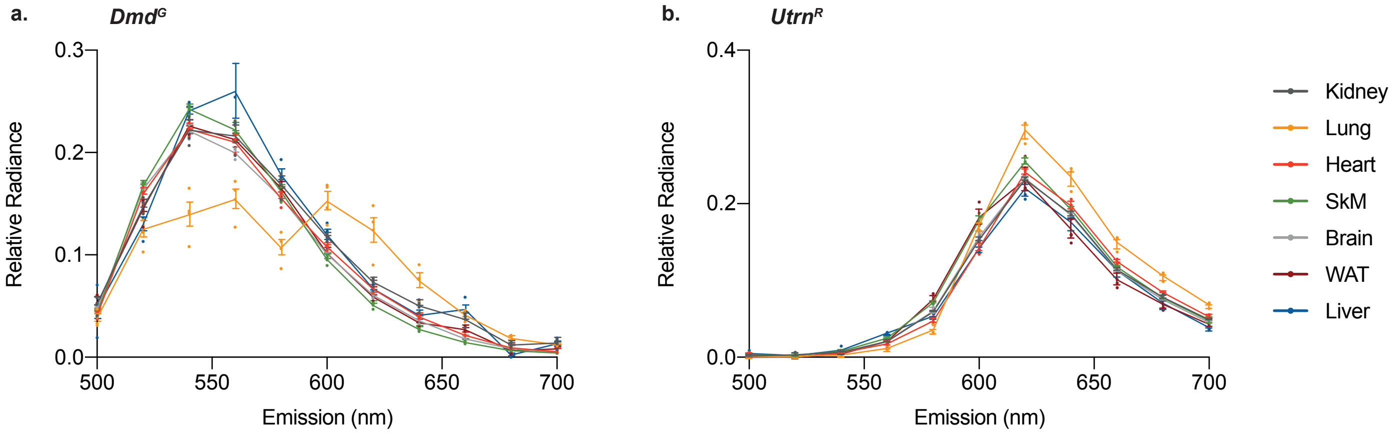

a.
DmdG
b.
UtrnR
Relative Radiance
Emission (nm)
0.0
0.1
0.2
0.3
0.4
500
550
600
650
700
Kidney
Lung
Heart
SkM
Brain
WAT
Liver

Supplementary Figure 2: Emission spectra of tissues dissected from *Dmd*^{G} and *Utrn*^{R} mice Related to Figure 2

Tissues dissected from single strain *Dmd*^{G} (**a**) or *Utrn*^{R} (**b**) reporter mice were imaged consecutively across emission filters between 500 and 700 nm. The graphs show the tissue-specific emission spectrum. For each tissue radiance was normalised against the open filter value for that tissue. Points show mean (n=3) +/- SEM. The spectra generated in (**a**) and (**b**) were used as reference spectra for the spectral unmixing analysis shown in Figure 2d-e.

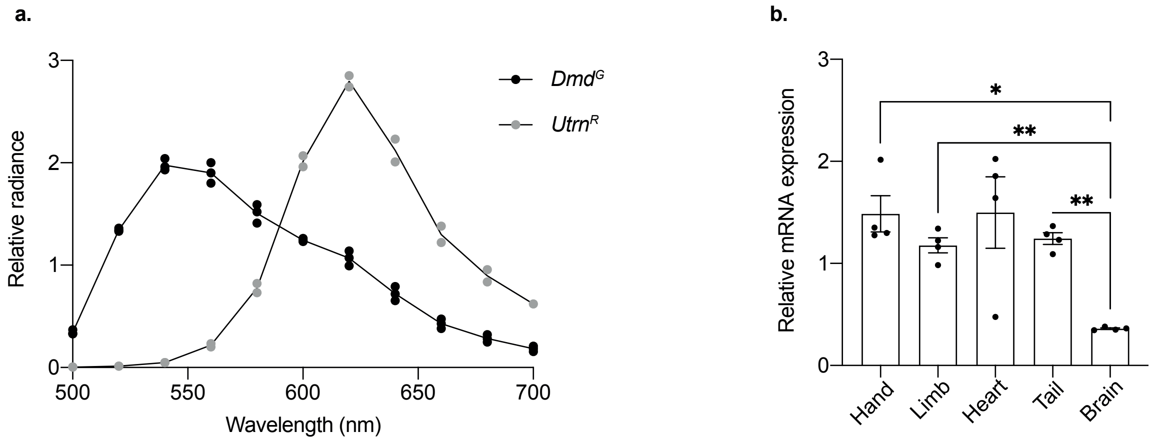

a.
Relative radiance
Wavelength (nm)
500
550
600
650
700
0
1
2
3
DmdG
UtrnR
b.
Relative mRNA expression
0
1
2
3
*
**
**
Hand
Limb
Heart
Tail
Brain

Supplementary Figure 3: Embryonic expression of *Utrn*

Related to Figure 3

a. Emission spectra of single strain *Dmd*^{G} (black) and *Utrn*^{R} (grey) E13.5 embryos. Consecutive images were taken using emission filters between 500 and 700 nm, graph shows quantification of whole-embryo radiance from each filter. *Dmd*^{G} emission peaked at 540 nm and *Utrn*^{R} emission peaked at 620 nm. These spectra were used as references for the spectral unmixing analysis shown in Figure 3d. **b.** RT-qPCR for *Utrn* expression in tissues dissected from E13.5 embryos. *Utrn* expression was high in the hands, limb, heart and tail but low in the brain. Expression levels were normalised to *18S* and *Tbp*. Bars show mean (n=4) +/- SEM, comparison between tissues was performed using a one-way ANOVA ($p=0.03$) with Tukey's multiple comparisons test (adjusted p values are shown: $^{*}p<0.05$, $^{**}p<0.01$).

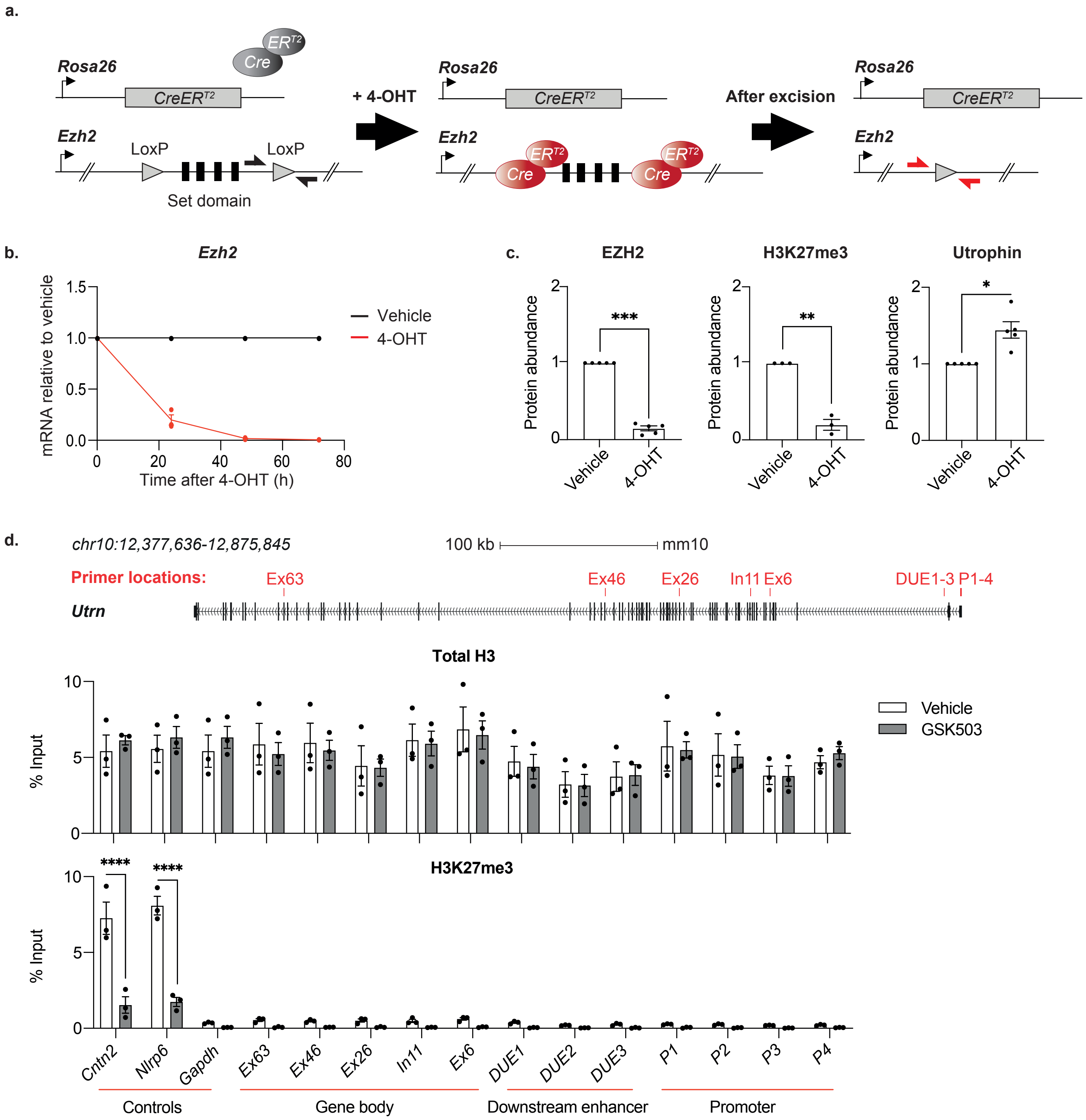

a.
Rosa26
CreER^T2
Cre
ER^T2
Ezh2
LoxP
LoxP
Set domain
+ 4-OHT
After excision
b.
Ezh2
mRNA relative to vehicle
Time after 4-OHT (h)
Vehicle
4-OHT
c.
EZH2
H3K27me3
Utrophin
Protein abundance
Vehicle
4-OHT
***
**
*
d.
chr10:12,377,636-12,875,845
100 kb
mm10
Primer locations:
Ex63
Ex46
Ex26
In11
Ex6
DUE1-3
P1-4
Utrn
Total H3
% Input
Vehicle
GSK503
H3K27me3
****
****
Cntn2
Nlrp6
Gapdh
Ex63
Ex46
Ex26
In11
Ex6
DUE1
DUE2
DUE3
P1
P2
P3
P4
Controls
Gene body
Downstream enhancer
Promoter

Supplementary Figure 4: Conditional knock out of *Ezh2* increased utrophin expression levels

Related to Figure 4

a. Schematic illustrating the conditional deletion strategy for *Ezh2*$^{flx/flx}$ in mice and myoblasts. The SET domain of endogenous *Ezh2* is flanked by *LoxP* sites and *CreER*^{t2} is constitutively expressed under the control of the *Rosa 26* locus. Upon 4-OHT treatment CRE-ER^{t2} translocates to the nucleus, binds to the *LoxP* sites and excises the *SET* domain. This translates into a non-functional EZH2 protein. Primers used to confirm *Ezh2* knock out are shown as black (WT allele) and red (excised allele) arrows. **b.** *Ezh2* mRNA levels in E22 myoblasts after 24-72 h 200 nM 4-OHT treatment by RT-qPCR; *Ezh2* was undetectable after 48 h. Expression levels were normalised to *18S* and *Tbp*. Points show mean (n=3) relative to the vehicle +/- SEM. **c.** Quantification of western blot of E22 myoblasts after 72 h 200 nM 4-OHT treatment (blots shown in Figure 4f). Protein was normalised to GAPDH, shown relative to vehicle treated control, with one-way paired t-tests for comparison (*p<0.05, **p<0.01, ***p<0.001). **d.** ChIP-qPCR for total histone H3 (upper) and H3K27me3 (lower) across the *Utrn* locus in U22 A5 myoblasts, before and after treatment with the EZH2 inhibitor GSK503 (10 µM). Primer locations shown above (Ex=exon, In=Intron, P=promoter, DUE=downstream *Utrn* enhancer). H3K27me3 was minimally detected across the *Utrn* locus (gene body, promoter and enhancer) compared to control regions (*Cntn2, Nlrp6*). Bars show mean percentage input +/- SEM. A two-way ANOVA with Sidak's multiple comparisons test was used to compare vehicle with GSK503 (adjusted p values are shown: **** p<0.0001).

a. ***Dystrophin (Dmd)***

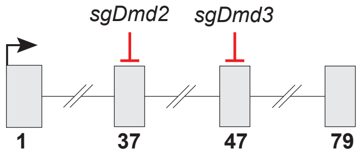

b.

Clone	Guide RNA	Mutation
2b8	*sgDmd2*	*WT / WT*
2a6	*sgDmd2*	*+1nt / +1nt*
3a7	*sgDmd3*	*WT / WT*
3b2	*sgDmd3*	*-4nt / -10nt*

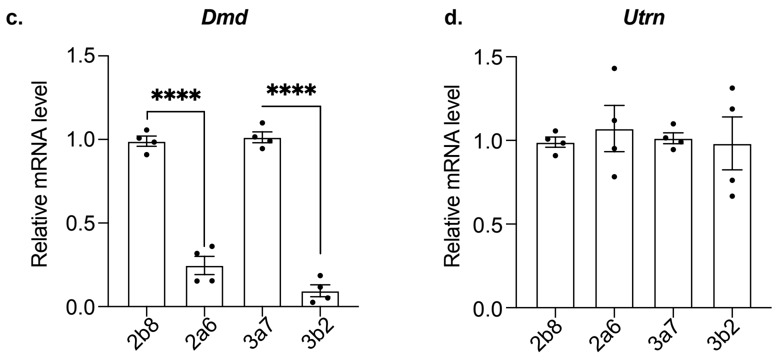

Supplementary Figure 5: Knock out of *Dmd* in *Utrn*$^{R+/-}$ myoblasts

Relative to Figure 5

a. Schematic of the mouse *Dmd* locus showing the positions of exons and the guide RNAs (*sgDmd2* and *sgDmd3*) used to create mutations using CRISPR/Cas9 in U22 A5 myoblasts. After sequencing, clones were selected that disrupt dystrophin expression by addition of deletion of nucleotides (nt) as shown in (**b**). **c.** RT-qPCR for *Dmd* (**c**) and *Utrn* (**d**) mRNA expression in WT (2b8, 3a7) and homozygous *Dmd* mutant clones (2a6, 3b2). Significantly less *Dmd* was produced in the mutant clones as a result of nonsense-mediated decay, while *Utrn* expression remained unaltered. Expression levels were normalised to *18S* and *Tbp* and shown relative to the corresponding WT clone. Bars show mean +/- SEM with paired t-tests performed to compare WT to knock out clones (*p<0.05, **p<0.01, ***p<0.001).

Supplementary Table 1: List of compounds tested in bioluminescence drug screen Corresponding to Figure 4

Full list of compounds used in bioluminescence drug screen shown in Figure 4b. Values refer to pIC50, unless otherwise stated.

	Name	Molecular Target	pIC50	Selectivity
**1**	Romidepsin	HDAC	HDAC1=7.44<br>HDAC2= 7.33,<br>HDAC4= 6.29,<br>HDAC6= 5.85	
**2**	GSK195	HDAC, class IIa	HDAC9=8.04,<br>HDAC7=7.33,<br>HDAC5=6.97,<br>HDAC4=6.95	HDAC8= 4.93, HDAC6= 4.32, (HDAC-1,-2,-3,-10, -11) <4
**3**	BIX01294	G9a	5.57	4.42 for GLP, no significant activity observed for histone methyltransferases
**4**	UNC0642	G9a/GLP	G9a and GLP= 8.6	>300-fold selective for G9a and GLP for all others tested
**5**	Chaetocin	SU(VAR)3-9	6.1	
**6**	GSK343	EZH2	8.4	60-fold selective vs EZH1, >1,000-fold for all others tested
**7**	GSK503	EZH2	8.3-8.7	200-fold selective vs EZH1, >10,000-fold selective for all others tested
**8**	UNC1999	EZH1/2	EZH1= 8.7,<br>EZH2= 7.35	>1000-fold selectivity over those tested
**9**	PRT4165	Bmi1/Ring1A	5.41	Also inhibits PRC1-mediated H2A ubiquitylation
**10**	MS37452	CBX7	Kd= 27.7 μM	
**11**	GSK2922801A	BAZ2A/B	BAZ2A Kd= 257 nM,<br>BAZ2B Kd= 136 nM	BRD9 Kd = 1.2 μM, TAF1 Kd = 3.2 μM, MT-1 (4%, CEREP)
**12**	GSK858	Pan BET	BRD4 BD1 7.6,<br>BRD4 BD2 8.2	
**13**	GSK151A	Pan BET	~7	CREBBP = 5.5
**14**	GSK726	Pan BET	BRD4 BD1 7.8,<br>BRD4 BD2 8.2	>100 fold over nearest non-BET
**15**	GSK602	BRD9	7.3	5.3 at BD1
**16**	GSK853	BRPF1	8.1	>1600-fold over all other Brds.<br>Brd4 1/2: 4.7/4.3
**17**	GSK311	BRPF1	6	<4.3 at Brd4 1/2
**18**	GSK959	BRPF1	7.1	>100-fold over all other Brds<br>Brd4 1/2: 4.7/4.6
**19**	GSK814	ATAD2	7.3	4.5 at BRD4 BD1
**20**	GSK077	CREBBP/ EP300	Kd= 151 nM (ITC)	Brd4-BD1 (ITC 5.6 μM)
**21**	GSK-J4	JMJD3	RFMS 4.8	EGLN 4.9, D2d 4.6
**22**	GSK854	LSD1	7.8	Selective vs LSD2 and other FAD utilizing enzymes
**23**	GSK591	PRMT5	8.3	PRMT5 selective, no other activities in methyltransferase panel

**24**	GSK712A	PRMT1	8.52	
**25**	UNC0379	SETD8	8.1	High selectivity over other methyltransferases
**26**	SGC0946	DOT1L	9.52	>100-fold selective over other histone methyltransferases/HMTs tested
**27**	U0126	MEK1/2	MEK1= 7.15, MEK2= 7.22	100-fold higher affinity for MEK than PD98059
**28**	LY3009120 (LY3009)	Pan Raf	A-Raf= 7.36, B-Raf = 7.5-7.4, C-Raf= 7.4	
**29**	LY3214996 (LY32)	ERK1/2	both= 8.3	
**30**	Ravoxertinib (Ravox)	ERK1/2	ERK1= 8.96, ERK2 = 9.52	
**31**	SB-747651A	MSK1	7.96	Also inhibits PRK2, RSK1, p70S6K and ROCK-II
**32**	OTS514 hydrochloride	TOPK	8.59	
**33**	OTS964	TOPK and CDK11	TOPK= 7.55, CDK11, Kd = 40 nM	
**34**	HI-TOPK-032	TOPK		
**35**	Hesperadin	Aurora B	6.6	Also reduces the activity of AMPK, Lck, MKK1, MAPKAP-K1, CHK1, and PHK at 1 μM
**36**	KN-62	CaMKII	Ki= 0.9 μM	
**37**	CX-4945	CK2	9	
**38**	Alsterpaullone	CDK and GSK-3	CDK1/cyclin B= 7.46, CDK2/cyclin A= 7.82, CDK2/cyclin E= 6.7, CDK5/p35= 7.4, GSK-3α/ β= 8.4	
**39**	Saracatinib	Src family	c-Src, Lck, c-YES, Lyn, Fyn, Fgr, and Blk = 8.57 - 7.96	
**40**	NVP-2	CDK9	9.29	
**41**	Chelerythrine Chloride	PKC	6.18	

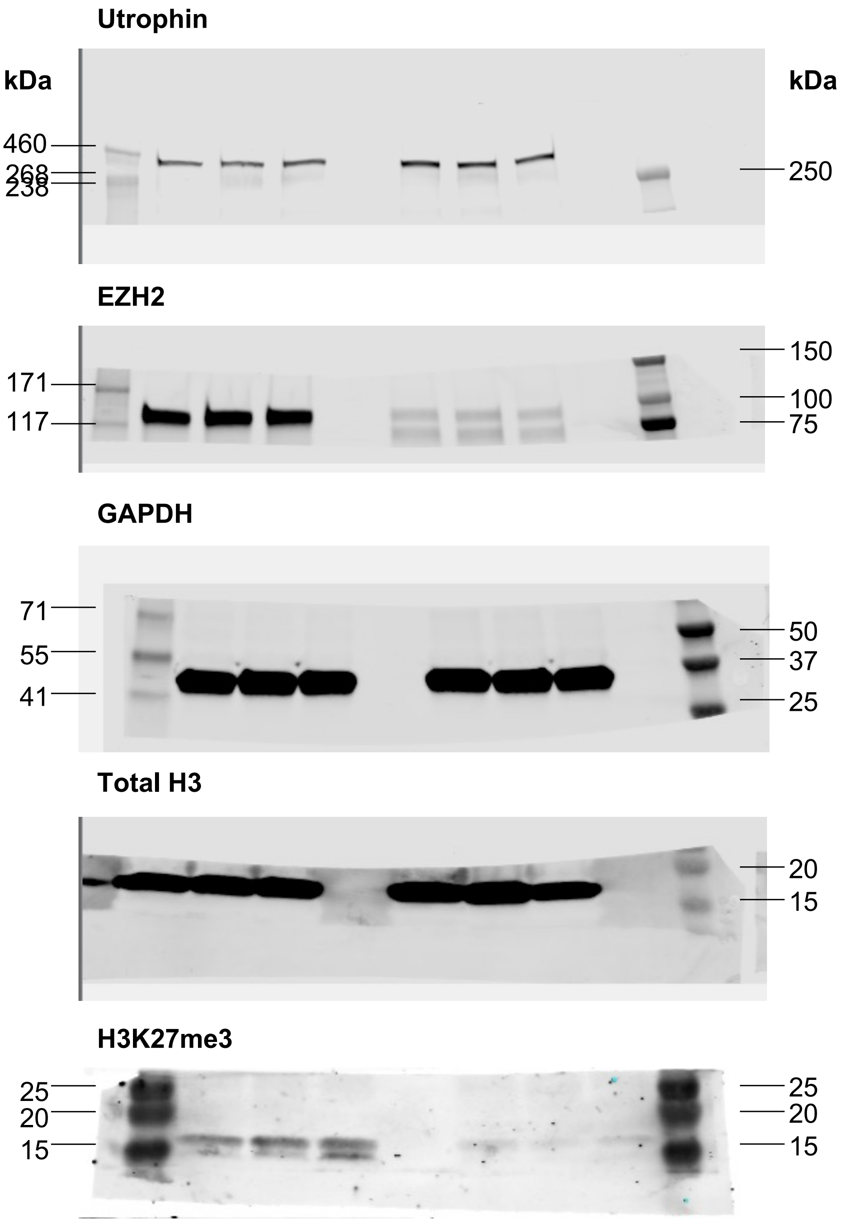

Supplementary Figure 6: Source Data for Figure 4f

Full images of the western blot gels shown in Figure 4f. Sizes of molecular weight markers (kDa) are shown alongside the blots
