# Supplementary material for: Endogenous bioluminescent reporters reveal a sustained increase in utrophin gene expression upon EZH2 and ERK1/2 inhibition
Source: Commun Biol. 2023 Mar 25;6:318. doi: 10.1038/s42003-023-04666-9 (PMC10039851; doi:10.1038/s42003-023-04666-9)
Supplement: Supplementary file 3 — Description of Additional Supplementary Files [file 42003_2023_4666_MOESM3_ESM.pdf]

Description of Additional Supplementary Files

File name: Supplementary Data 1

Description: The source data behind all graphs in the paper

File name: Supplementary Video 1

Description: Utrophin expression in mid-gestation, visualised by optical projection tomography. Related to Figure 3e. Video showing X-Gal staining of lacZ in UtrnR E13.5 embryos as visualised by OPT imaging. Red signal represents lacZ detection with Page 7 of 17 The image part with relationship ID rId1 was not found in the file. greyscale showing fluorescent reconstruction of whole sample volume.
