# Supplementary material for: Endogenous bioluminescent reporters reveal a sustained increase in utrophin gene expression upon EZH2 and ERK1/2 inhibition
Source: Commun Biol. 2023 Mar 25;6:318. doi: 10.1038/s42003-023-04666-9 (PMC10039851; doi:10.1038/s42003-023-04666-9)
Supplement: Supplementary file 6 — Reporting Summary [file 42003_2023_4666_MOESM6_ESM.pdf]

nature portfolio

Corresponding author(s): Amanda G. Fisher

Last updated by author(s): 2023.02.22

Reporting Summary

Nature Portfolio wishes to improve the reproducibility of the work that we publish. This form provides structure for consistency and transparency in reporting. For further information on Nature Portfolio policies, see our Editorial Policies and the Editorial Policy Checklist.

Statistics

For all statistical analyses, confirm that the following items are present in the figure legend, table legend, main text, or Methods section.

n/a	Confirmed	
☐	☒	The exact sample size (*n*) for each experimental group/condition, given as a discrete number and unit of measurement
☐	☒	A statement on whether measurements were taken from distinct samples or whether the same sample was measured repeatedly
☐	☒	The statistical test(s) used AND whether they are one- or two-sided<br>*Only common tests should be described solely by name; describe more complex techniques in the Methods section.*
☐	☒	A description of all covariates tested
☐	☒	A description of any assumptions or corrections, such as tests of normality and adjustment for multiple comparisons
☐	☒	A full description of the statistical parameters including central tendency (e.g. means) or other basic estimates (e.g. regression coefficient) AND variation (e.g. standard deviation) or associated estimates of uncertainty (e.g. confidence intervals)
☐	☒	For null hypothesis testing, the test statistic (e.g. *F*, *t*, *r*) with confidence intervals, effect sizes, degrees of freedom and *P* value noted<br>*Give P values as exact values whenever suitable.*
☒	☐	For Bayesian analysis, information on the choice of priors and Markov chain Monte Carlo settings
☒	☐	For hierarchical and complex designs, identification of the appropriate level for tests and full reporting of outcomes
☒	☐	Estimates of effect sizes (e.g. Cohen's *d*, Pearson's *r*), indicating how they were calculated

Our web collection on statistics for biologists contains articles on many of the points above.

Software and code

Policy information about availability of computer code

Data collection	• IVIS images were generated using an IVIS Spectrum (Perkin Elmer) and the Living Image software (version 4.3.1).<br>• The Bio-Rad CFX96 system was used to acquire qPCR data<br>• Western blot gels were imaged on a LICOR Odyssey imaging machine.<br>• X-gal staining of frozen tissue sections was visualised using an Axio.Scan.Z1 Slide Scanner (Zeiss).<br>• OPT images were recorded using a CCD camera operated at 2×2-pixel binning (Zyla 5.5, Andor Technology Ltd), images were acquired every 0.9° over a full 360° sample rotation through a 708±37 nm band-pass filter (FF01-708/75-25, Laser 2000 UK Ltd).
Data analysis	• IVIS images were analysed using the Living Image software (version 4.5.2) (Perkin Elmer). Spectral unmixing analysis was performed using the manual setting.<br>• qPCR results were analysed using Microsoft Excel and GraphPad Prism (version 8).<br>• Western blot results were analysed using the Image Studio Lite software.

For manuscripts utilizing custom algorithms or software that are central to the research but not yet described in published literature, software must be made available to editors and reviewers. We strongly encourage code deposition in a community repository (e.g. GitHub). See the Nature Portfolio guidelines for submitting code & software for further information.

Data

Policy information about availability of data

All manuscripts must include a data availability statement. This statement should provide the following information, where applicable:

- Accession codes, unique identifiers, or web links for publicly available datasets
- A description of any restrictions on data availability
- For clinical datasets or third party data, please ensure that the statement adheres to our policy

Provide your data availability statement here.

Human research participants

Policy information about studies involving human research participants and Sex and Gender in Research.

Reporting on sex and gender	*Use the terms sex (biological attribute) and gender (shaped by social and cultural circumstances) carefully in order to avoid confusing both terms. Indicate if findings apply to only one sex or gender; describe whether sex and gender were considered in study design whether sex and/or gender was determined based on self-reporting or assigned and methods used. Provide in the source data disaggregated sex and gender data where this information has been collected, and consent has been obtained for sharing of individual-level data; provide overall numbers in this Reporting Summary. Please state if this information has not been collected. Report sex- and gender-based analyses where performed, justify reasons for lack of sex- and gender-based analysis.*
Population characteristics	*Describe the covariate-relevant population characteristics of the human research participants (e.g. age, genotypic information, past and current diagnosis and treatment categories). If you filled out the behavioural & social sciences study design questions and have nothing to add here, write "See above."*
Recruitment	*Describe how participants were recruited. Outline any potential self-selection bias or other biases that may be present and how these are likely to impact results.*
Ethics oversight	*Identify the organization(s) that approved the study protocol.*

Note that full information on the approval of the study protocol must also be provided in the manuscript.

Field-specific reporting

Please select the one below that is the best fit for your research. If you are not sure, read the appropriate sections before making your selection.

☒ Life sciences ☐ Behavioural & social sciences ☐ Ecological, evolutionary & environmental sciences

For a reference copy of the document with all sections, see nature.com/documents/nr-reporting-summary-flat.pdf

Life sciences study design

All studies must disclose on these points even when the disclosure is negative.

Sample size	For all experiments, sample size was chosen to ensure accurate, reproducible results. Mouse IVIS imaging experiments involved a minimum of 3 mice per group, with data obtained from a minimum of 2 separate litters and images. Mouse tissue for qRT-PCR experiments was dissected from 3 mice. X-gal staining was performed twice with similar results, only one example is shown. Spectral unmixing experiments were performed in triplicate, to accurately observe the trend of the bioluminescence spectra for each genotype. This study did not involve any external intervention to the mice and the animals used were of equivalent ages and housed identically, therefore, we deemed these sample sizes enough to produce reproducible results whilst minimising the number of animals sacrificed, in accordance with the NC3Rs.<br><br>Cell culture drug treatment and differentiation experiments were performed in a minimum of triplicate. The cells and culture conditions used was kept consistent across experiments and so we determined that this was enough to ensure accurate, reproducible results and perform statistical analysis. RT-qPCR experiments were performed in technical triplicate to account for pipetting errors.
Data exclusions	The only data excluded from this study were a small number of technical replicates from RT-qPCR analysis. In this case, each biological sample was run in triplicate wells, should one well deviate substantially from the other two wells then it was removed from the analysis and a mean determined from the remaining two samples. Biological replicates were not excluded.
Replication	All experiments were performed in replicate to confirm the reproducibility of the findings, in all cases this was successful.<br><br>To maintain the reproducibility of findings, all animals used in this study were maintained using the same conditions and housed in the same animal holding room. This means that diet, microbiota, light/dark cycles, temperature and cage enrichment will all be equivalent. All animals used in this study were between 3-10 weeks old, and within individual experiments the age difference never exceeded 1 week. Other experimental parameters such as time between D-Luc exposure and imaging for IVIS experiments and conditions of RNA extraction from

tissues were determined initially and maintained across experimental replicates to ensure consistency.

Similarly, for cell culture experiments, the culture conditions and passage number of the cells used was maintained as consistent as possible throughout.

Randomization	For the animal experiments performed in this study, randomisation was not relevant as the tested variable was the genotype of the animal.<br><br>Likewise, for cell culture experiments involving drug tested vs vehicle tested samples the cells were split from the same initial vial, grown in equivalent conditions prior to treatment and, with the exception of the treatment, all conditions during treatment were identical for both samples. Therefore, randomization was not required.
Blinding	For mouse adult/embryo experiments involving mixed genotypes, researchers were blinded to the genotypes of the animals involved during data collection. For bioluminescence drug treatment experiments involving myoblasts, researchers were not blinded, this is because the location of the control sample needs to be known to determine that the imaging is successful and as an essential part of the data analysis.

Reporting for specific materials, systems and methods

We require information from authors about some types of materials, experimental systems and methods used in many studies. Here, indicate whether each material, system or method listed is relevant to your study. If you are not sure if a list item applies to your research, read the appropriate section before selecting a response.

Materials & experimental systems

n/a	Involved in the study	
☐	☒	Antibodies
☐	☒	Eukaryotic cell lines
☒	☐	Palaeontology and archaeology
☐	☒	Animals and other organisms
☒	☐	Clinical data
☐	☐	Dual use research of concern

Methods

n/a	Involved in the study	
☒	☐	ChIP-seq
☒	☐	Flow cytometry
☒	☐	MRI-based neuroimaging

Antibodies

Antibodies used	• Utrophin, Mouse monoclonal to Utrn 8A4, Santa Cruz, sc-33700<br>• EZH2, Mouse monoclonal to 11/EZH2, BD, 612666<br>• Total H3, Rabbit polyclonal to Histone H3, Abcam, ab1791<br>• GAPDH, Mouse monoclonal [6C5] to GAPDH, Abcam, ab8245<br>• H3K27me3, Mouse monoclonal to tri methyl K27, Abcam, ab6002
Validation	• Utrophin: as shown on the datasheet for sc-33700, the antibody was shown to detect Utrophin in mouse C2C12 myoblasts<br>• EZH2: The antibody was used to detect Ezh2 by western blotting for mouse samples in a number of previously published works (Zhang, H., et al. Nat Commun 8, 14922 (2017); Arvey, A., et al. Nat Immunol 15, 580–587 (2014); Zhang H, Cancer Discov. 9 1006-21 (2016)). Furthermore, as shown in Figure 4F of the current study, upon Ezh2 conditional knock out a significant decrease in signal was detected using the Ezh2 antibody BD 612666.<br>• Total H3: Antibody ab1791 is covered by the Abcam Abguarantee for detection of total H3 by ChIP and western blot, examples of which are shown on the webpage: the antibody has been used for western blot of mouse skeletal muscle, with signal only detected in the nuclear fraction; likewise, the website shows use of the antibody for ChIP of mouse samples.<br>• Gapdh: Antibody ab8245 is covered by the Abcam Abguarantee for detection of Gapdh by western blot, an example of which is shown on the webpage.<br>• H3K27me3: Antibody ab6002 is covered by the Abcam Abguarantee for detection of H3K27me3 by western blot and ChIP, examples of which are shown on the webpage.

Eukaryotic cell lines

Policy information about cell lines and Sex and Gender in Research

Cell line source(s)	• Q2TSA583, a fibroblast cell line generated by Jat et al., 1991, stably expresses SV40 TAg and was used in the generation of the two cell lines described in this study.<br>• U22 A5, myoblasts generated for this paper from a 22-day old female Utrn-RFluc-LacZ (UtrnR) mouse and immortalised using the simian virus 40 (SV40) large T-antigen (TAg) isolated from the TAg producing cell line Q2TSA583.<br>• E22, myoblasts generated for this paper from a 22-day old male EZH2flx/flxRosa26ERt2–Cre/ERt2–Cre (Ezh2flx/flx) and immortalized using TAg isolated from Q2TSA583.
Authentication	TAg genetics and expression in Q2TSA583 cells was confirmed using PCR and qRT-PCR.
Mycoplasma contamination	All cell lines used in this study tested negative for mycoplasma.
Commonly misidentified lines<br>(See ICLAC register)	*Name any commonly misidentified cell lines used in the study and provide a rationale for their use.*

Animals and other research organisms

Policy information about studies involving animals; ARRIVE guidelines recommended for reporting animal research, and Sex and Gender in Research

Laboratory animals	Mice lines involved in this study:<br>• Utrn-RFluc-LacZ (UtrnR), generated by OzGene, C57BL6/Albino<br>• Dmd-CBG99Luc (DmdG), generated by Taconic Biosciences, C57BL6/Albino<br>• Utrn-RFluc-LacZ x Dmd-CBG99Luc (Dual or DmdG x UtrnR), generated in house by crossing the above two lines, C57BL6/Albino<br>• EZH2flx/flxRosa26ERt2–Cre/ERt2–Cre (Ezh2flx/flx), a gift from A. Tarakhovsky (The Rockefeller University).<br>Mice were housed on a 12-hour light-dark cycle with a temperature range of 21 +/- 2 C and humidity range of 55 +/- 10 % in pathogen free conditions. All experiments were performed on mice aged between 3-10 weeks old.
Wild animals	No wild animals were involved in this study
Reporting on sex	Dystrophin is an X-linked gene, therefore for studies involving heterozygous DmdG mice only female mice were used, so one WT and one KI allele were present.
Field-collected samples	No field-collected samples were involved in this study
Ethics oversight	All animal procedures were performed in accordance with the British Home Office Animal (Scientific Procedures) Act 1986 and the GSK Policy on the Care, Welfare and Treatment of Animals. The mouse work was approved by the Imperial College AWERB committee and performed under a UK Home Office Project License and Personal Licenses

Note that full information on the approval of the study protocol must also be provided in the manuscript.

Dual use research of concern

Policy information about dual use research of concern

Hazards

Could the accidental, deliberate or reckless misuse of agents or technologies generated in the work, or the application of information presented in the manuscript, pose a threat to:

No	Yes	
☒	☐	Public health
☒	☐	National security
☒	☐	Crops and/or livestock
☒	☐	Ecosystems
☒	☐	Any other significant area

Experiments of concern

Does the work involve any of these experiments of concern:

No	Yes	
☒	☐	Demonstrate how to render a vaccine ineffective
☒	☐	Confer resistance to therapeutically useful antibiotics or antiviral agents
☒	☐	Enhance the virulence of a pathogen or render a nonpathogen virulent
☒	☐	Increase transmissibility of a pathogen
☒	☐	Alter the host range of a pathogen
☒	☐	Enable evasion of diagnostic/detection modalities
☒	☐	Enable the weaponization of a biological agent or toxin
☒	☐	Any other potentially harmful combination of experiments and agents
